# Biotechnology Revolution Shaping the Future of Diabetes Management

**DOI:** 10.3390/biom14121563

**Published:** 2024-12-07

**Authors:** Nilima Rajpal Kundnani, Bogdan Lolescu, Anca-Raluca Dinu, Delia Mira Berceanu-Vaduva, Patrick Dumitrescu, Tudor-Paul Tamaș, Abhinav Sharma, Mihaela-Diana Popa

**Affiliations:** 1 Department of Cardiology—Internal Medicine and Ambulatory Care, Prevention and Cardiovascular Recovery, “Victor Babeș” University of Medicine and Pharmacy, 300041 Timisoara, Romania; knilima@umft.ro (N.R.K.);; 2 Research Centre of Timisoara Institute of Cardiovascular Diseases, “Victor Babeșs” University of Medicine and Pharmacy, 300041 Timisoara, Romania; 3 Doctoral School Medicine-Pharmacy, “Victor Babeș” University of Medicine and Pharmacy from Timisoara, Eftimie Murgu Sq. No. 2, 300041 Timisoara, Romania; 4 Department XVI, Medical Recovery, “Victor Babeş” University of Medicine and Pharmacy, 300041 Timisoara, Romania; 5 Research Center for Assessment of Human Motion and Functionality and Disability, “Victor Babeșs” University of Medicine and Pharmacy, Eftimie Murgu Square, No. 2, 300041 Timisoara, Romania; 6 “Pius Brinzeu” Emergency Clinical County Hospital, Bld Liviu Rebreanu, No. 156, 300723 Timisoara, Romania; 7 Discipline of Microbiology, Department XIV Microbiology, University of Medicine and Pharmacy from Timisoara, Eftimie Murgu Sq. No. 2, 300041 Timisoara, Romania; berceanu.delia@umft.ro (D.M.B.-V.);; 8 Faculty of Medicine, University of Medicine and Pharmacy from Timisoara, Eftimie Murgu Sq. No. 2, 300041 Timisoara, Romania; 9 Discipline of Physiology, Department III—Functional Sciences, University of Medicine and Pharmacy from Timisoara, Eftimie Murgu Sq. No. 2, 300041 Timisoara, Romania

**Keywords:** pancreas, insulin, recombinant peptides, GLP-1 receptor (GLP1R) agonists, glucose-dependent insulinotropic polypeptide (GIP) receptor agonists

## Abstract

Introduction: Diabetes mellitus (DM) has a millennia-long history, with early references dating back to ancient Egypt and India. However, it was not until the 20th century that the connection between diabetes and insulin was fully understood. The sequencing of insulin in the 1950s initiated the convergence of biotechnology and diabetes management, leading to the development of recombinant human insulin in 1982. This marked the start of peptide-based therapies in DM. Recombinant peptides for DM treatment: Numerous recombinant peptides have been developed since, starting with modified insulin molecules, with the aim of bettering DM management through fine-tuning the glycemic response to insulin. Peptide-based therapies in DM have expanded substantially beyond insulin to include agonists of Glucagon-like peptide-1 receptor and Glucose-dependent insulinotropic polypeptide receptor, glucagon receptor antagonists, and even peptides exerting multiple receptor agonist effects, for better metabolic control. Insulin pumps, continuous glucose monitoring, and automated insulin delivery systems: The development of modern delivery systems combined with real-time glucose monitoring has significantly advanced diabetes care. Insulin pumps evolved from early large devices to modern sensor-augmented pumps with automated shutoff features and hybrid closed-loop systems, requiring minimal user input. The second-generation systems have demonstrated superior outcomes, proving highly effective in diabetes management. Islet cell transplantation, organoids, and biological pancreas augmentation represent innovative approaches to diabetes management. Islet cell transplantation aims to restore insulin production by transplanting donor beta cells, though challenges persist regarding graft survival and the need for immunosuppression. Organoids are a promising platform for generating insulin-producing cells, although far from clinical use. Biological pancreas augmentation relies on therapies that promote beta-cell (re)generation, reduce stress, and induce immune tolerance. Further biotechnology-driven perspectives in DM will include metabolic control via biotechnology-enabled tools such as custom-designed insulin hybrid molecules, machine-learning algorithms to control peptide release, and engineering cells for optimal peptide production and secretion.

## 1. Introduction

Diabetes mellitus (DM), a condition characterized by hyperglycemia due to insulin underproduction and/or insulin resistance, has been documented for millennia. The earliest references to what is now recognized as diabetes come from ancient Egypt and India. Around 1500 BCE, the “Ebers Papyrus” documented a disease that caused frequent urination and weight loss. In ancient India, diabetes was known as “Madhumeha”, which translates to “honey urine.” Around 500 BCE, Indian healers observed that insects were attracted to the urine of individuals suffering from a disease, indicating that the urine was sweet—an observation that foreshadowed later scientific discoveries. During the Middle Ages, the medical knowledge in Europe regressed, while the Arab scholars preserved and expanded upon the writings of the Greeks and Romans. The Persian physician Avicenna (Ibn Sina) wrote about diabetes in his “Canon of Medicine” (1025 CE), noting the sweet taste of urine and associating the disease with both kidney disorders and excessive eating and drinking [1,2].

Later chemistry advances enabled new insights into diabetes. In 1674, Thomas Willis rediscovered the sweet taste of diabetic urine, which led to the understanding that diabetes was related to sugar metabolism, while Matthew Dobson’s experiments proved that diabetic serum and urine contained excess sugar. In 1815, the French chemist Michel Eugène Chevreul identified the carbohydrate in diabetic urine as glucose. Thus, the definitive connection between diabetes and hyperglycemia was made. Around the same time, Claude Bernard, a French physiologist, demonstrated the role of the liver in glucose metabolism [1,3,4].

The connection between the pancreas and diabetes was established in 1869, when the medical student Paul Langerhans discovered clusters of cells in the pancreas, later known as the islets of Langerhans. In 1889, two German physicians, Oskar Minkowski and Josef von Mering, removed the pancreas from a dog and observed that it developed diabetes. This discovery implied that the pancreas produced a substance vital for glucose regulation, though insulin had not yet been identified. By the early 20th century, several scientists were focused on identifying the pancreatic factor that regulated glycemia, as their efforts were hampered by pancreatic proteases digestion of the extract before it could be experimented upon [1].

The breakthrough came in 1921, when Frederick Banting, along with Charles Best, devised a method to isolate the hormone insulin. Working at the University of Toronto under the supervision of Professor John Macleod, Banting and Best found a way to prevent the lysis of the insulin-producing cells. They then extracted insulin and successfully treated diabetic dogs. The impact of insulin discovery combined with the political situation after the First World War led to the swift award of a Nobel Prize in Physiology or Medicine to Banting and Macleod in 1923, a decision that sparked controversy later on. Further developments led to better purification and chemical modifications of insulin that were more stable and longer acting. By the mid-20th century, insulin had become the standard DM treatment [2].

Insulin was the first peptide to be sequenced, by Frederick Sanger in the 1950s, an achievement that led to him being awarded the Nobel Prize in 1958. That was also the moment when biotechnology and diabetes mellitus developed a common history, as protein sequencing is now an important tool for engineering novel peptide-based therapies. Another Nobel Prize was shared in 1977 by Rosalyn Yalow, Roger Guillemin, and Andrew Schally, who developed a radioimmunoassay for insulin and other peptide hormones. This significant record for the scientists working to understand diabetes mechanisms only heralded the dawn of the modern age in disease treatment through bioengineering peptides, initially produced in *E. coli*, by recombinant DNA techniques. Human recombinant insulin became the first such drug, approved for human use in 1982 [5,6,7,8,9].

## 2. Recombinant Peptides for DM Treatment

Naturally, the first therapeutic peptide candidate for recombinant production was the human insulin, with its peptide sequence as described by Sanger a couple of decades before. This marked a major milestone in the use of *E. coli* as a host organism for the production of relatively complex proteins like insulin, which requires proper oxidative folding as it is a heterodimer (composed of two different peptide chains). *E. coli* was proven to be a versatile organism, due to its rapid growth, high protein synthesis yield, and cost-effectiveness. Still, the absence of post-translational modifications, such as glycosylation, limit its usefulness for the production of more complex recombinant biopharmaceuticals [10,11,12].

A clinical pharmacological study of the recombinant insulin on healthy volunteers concluded in 1982 that human insulin produced by recombinant DNA technology showed similar pharmacokinetic and pharmacological profiles to porcine insulin, making it a viable alternative for clinical use. A slight advantage of human insulin in terms of bioavailability after subcutaneous injection was noticed for its potential clinical relevance for long-term therapy. Additionally, there were no significant adverse effects associated with either form of insulin, supporting the safety of recombinant human insulin, to be used in gestational DM, Type 1 DM, or as intermittent insulin therapy in Type 2 DM. This study aligned with previous research, affirming the safety and efficacy of recombinant human insulin in managing blood glucose levels. A frequent issue with animal-derived insulin was the enhanced immunogenicity of the impure preparations, with generation of anti-insulin antibodies, leading to insulin resistance and lipoatrophy in a significant number of cases. Yet even in the case of modern insulins, immune responses with low titers of IgG and IgE antibodies were still observed in some patients, where the antibody response could predict adverse events such as injection site reactions [13,14,15,16].

## 3. Overcoming the Drawbacks of Natural Insulin

The absorption of natural insulin after an injection can be inconsistent, based on local factors like the injection site, body fat, and blood flow. Furthermore, natural insulin tends to form hexamers at the injection site, which must break down into monomers before being efficiently absorbed into the bloodstream. This also delays the onset of action and can lead to inconsistent absorption rates when injected subcutaneously. It takes about 30–60 min for natural insulin to begin lowering glycemia after injection, which means that patients need to plan their insulin injections well before meals. This is not ideal for managing postprandial glucose spikes, which occur more rapidly. This variability leads to difficulties in predicting the insulin’s exact effect and timing, complicating glycemic control [17].

Furthermore, the kinetics of glucose absorption from the intestines into the bloodstream following carbohydrate ingestion typically occurs rapidly, with peak glucose levels observed within 30–60 min after a meal. This sharp rise necessitates a corresponding increase in insulin secretion to facilitate glucose uptake by tissues and to prevent hyperglycemia. However, early insulins, such as regular human insulin, had a delayed onset of action (30–60 min) and peaked at 2–4 h, which could not match the timing of postprandial glucose spikes. This mismatch led to suboptimal glycemic control, including postprandial hyperglycemia and delayed hypoglycemia as insulin activity persisted beyond the period of glucose absorption [18,19].

In order to address these issues, rapid-acting insulin analogs like insulin lispro (Humalog, Utrecht, The Netherlands) and insulin aspart (NovoLog, New Jersey, USA; NovoRapid, Bagsværd, Denmark) were designed to prevent hexamer formation. These analogs are absorbed more quickly after injection, with onset of action in 10–20 min, allowing for more flexibility with meal timing and better postprandial glycemic control. A derivative of insulin aspart, named faster aspart, incorporates two additional excipients—niacinamide and L-arginine—designed to accelerate insulin absorption after subcutaneous administration. Faster aspart significantly improved the onset and early exposure of insulin compared to insulin aspart, offering quicker glucose-lowering effects, especially within the first 30 min post-injection. This enhanced pharmacokinetic profile more closely mimics the body’s natural insulin secretion during meals, particularly beneficial for managing postprandial glycemia. Faster aspart has also demonstrated consistent improvement in glucose-lowering effects in comparison to standard insulin aspart, particularly in patients with Type 1 DM using basal-bolus therapy or continuous subcutaneous insulin infusion (CSII). While its ultra-fast action may pose a risk of hypoglycemia immediately following meals, it also reduces the risk of late postprandial hypoglycemia by better aligning insulin activity with glucose disposal. Moreover, faster aspart could offer flexibility for patients with irregular meal schedules, potentially allowing for post-meal insulin administration. However, further studies were recommended to explore optimal dosing schedules and long-term safety in diverse clinical settings, especially for individuals using insulin pumps and those with mixed meal patterns [20,21].

Insulin lispro-aabc achieved faster onset and earlier glucose-lowering effects than conventional insulin lispro, with trials showing reduced postprandial glucose (PPG) levels without significantly different hypoglycemia risks. Insulin lispro-aabc features added excipient treprostinil to accelerate absorption and reduce PPG excursions more effectively than older rapid-acting insulins. However, the faster acting insulins’ ability to improve glycated hemoglobin (HbA1c) reduction appears similar to traditional rapid-acting insulins, despite reductions in 1- and 2-hour PPG levels. Despite the short-term improvements, the long-term clinical outcomes of ultra-rapid-acting insulins, such as potential improvements in HbA1c control and hypoglycemia safety, appear to remain comparable to rapid-acting insulins [22].

The physiological secretion of insulin has a baseline level in the interprandial periods and during sleep, which would be difficult to obtain with recombinant insulins that are designed to compensate for the glycemic load after a meal, such as the above insulins. Thus, in contrast to insulin aspart and insulin lispro, which were designed to better mimic the body’s rapid insulin response to food intake, basal analogs such as insulin degludec (IDeg) provide ultra-long action, lasting up to 42 h and giving patients more flexibility in dosing time and reducing glycemic control variability. Its structure includes a fatty acid side chain that allows it to form stable multi-hexamer chains at the injection site, leading to a slow, steady release into the bloodstream. Its ultra-long duration and flat pharmacodynamic profile minimize the variability in glucose-lowering effects, especially compared to insulin glargine (IGlar), used as a comparator in many studies. Clinical trials have shown that IDeg offers a flexible dosing schedule without compromising glycemic control, a key benefit over other basal insulins, particularly in populations like the elderly or those with varying lifestyles. In terms of efficacy, IDeg has demonstrated non-inferiority to IGlar in reducing HbA1c levels. However, the most notable advantage of IDeg was its lower risk of hypoglycemia, particularly nocturnal hypoglycemia, which is a significant concern in insulin therapy. Additionally, patients switching to IDeg from other insulins would typically require a dose adjustment, especially when transitioning from a twice-daily basal insulin regimen. The ability to administer IDeg with flexible timing also contributes to patient convenience and adherence [23,24].

A clinical study also evaluated the combination of IDeg with rapid-acting insulin aspart (IDegAsp) and the fixed-ratio formulation with the Glucagon-like peptide-1 (GLP-1) analog liraglutide (IDegLira), both of which offer further options for simplifying treatment regimens in diabetes management. Moreover, IDeg has been shown to be cost-effective in several economic models, particularly due to the reduced risk of hypoglycemia and lower overall insulin requirements. Overall, insulin degludec was deemed to provide a clinically beneficial and patient-friendly option for managing diabetes, addressing many of the challenges associated with basal insulin therapy [23,25].

These insulin types together with others are summarized in Table 1.

Most recently, a designer glucose-sensitive insulin conjugate-NNC2215 was developed to reduce the risk of hypoglycemia in diabetes treatment. The key innovation in the peptide is its glucose-responsive switch, which modulates its bioactivity based on ambient glucose levels. This result was achieved by conjugating a glucose-binding macrocycle and glucoside to insulin, allowing the molecule to transition between active and less-active states depending on blood glucose concentrations. The study included in vitro and in vivo experiments to characterize NNC2215’s glucose sensitivity and pharmacological effects. In vitro, the compound exhibited a quasi-linear glucose-dependent increase in binding affinity for the human insulin receptor (hIR-A), between glucose concentrations of 3 and 20 mM, reflecting the glycemic range common in people with diabetes. In vivo studies in rats and pigs showed that NNC2215 attenuates hypoglycemia compared to non-glucose-sensitive insulin analogs, such as insulin degludec. NNC2215 also demonstrated its ability to activate appropriately in response to glucose challenges in diabetic rats, offering potential improvements in controlling postprandial glucose levels. Thus, NNC2215 could provide significant advantages in diabetes management by offering better glycemic control with a lower risk of hypoglycemia, especially during fasting or overnight periods when hypoglycemia is more likely to occur. The combination of its glucose-responsive behavior and protection against hypoglycemia suggests that NNC2215 could lead to more aggressive insulin titration without the fear of dangerously low glycemia. Previous approaches include MK-2640, an insulin-oligosaccharide conjugate with glucose-dependent receptor affinity, tested in a diabetic dog model, and polymer-based systems wherein insulin is encapsulated within a glucose-responsive polymeric matrix with controlled permeability for insulin. However, the polymer’s biocompatibility could pose an issue, with unknown long-term effects. These results prefigure future applications of molecular switches for autonomous control of insulin bioactivity based on fluctuating glucose levels [30,31,32].

## 4. Non-Insulin Peptide Treatments in DM

With the advances in biotechnology, several other peptide therapies have emerged to improve glycemic control, enhance insulin secretion, and manage DM-related complications. These therapies were particularly focused on addressing Type 2 DM by targeting other glucose regulation pathways [33,34,35,36,37,38,39,40,41,42,43].

GLP-1 receptor (GLP1R) agonists mimic the action of the naturally occurring incretin hormone GLP-1, which enhances the glucose-dependent insulin secretion, inhibits glucagon release, slows gastric emptying, and promotes satiety, which helps reduce food intake. Exenatide, a peptide originally derived from the saliva of the Gila monster, became the first GLP1R agonist to be developed in 2005, with both short-acting and long-acting formulations derived later. Other GLP1R agonists include liraglutide, a longer-acting peptide that can be administered once daily and has also been approved for weight loss therapy, together with dulaglutide and semaglutide, which are administered once weekly, a more convenient dosing schedule compared to earlier agents. Semaglutide was the first oral GLP1R agonist, as it is also became available as an oral formulation in 2019 [33,34,35,36].

GLP1R agonists not only promote glycemic control but also have significant cardiovascular benefits, with an anti-inflammatory role and weight-reducing effects. Studies have shown that they reduce the risk of major adverse cardiovascular events in people with Type 2 DM. Recently, these peptides have emerged as potential therapeutic agents in inflammatory bowel disease. A new study also demonstrated an in vivo increase in the number of human pancreatic beta cells transplanted into immunodeficient mice. This increase was induced by a combination treatment involving DYRK1A kinase inhibition and GLP1R agonists, thus proving the therapeutic potential and a favorable preclinical safety profile of the combination for diabetes treatment [36,37,38,39].

Furthermore, a small-scale retrospective analysis evaluated the effects of semaglutide in 10 adults newly diagnosed with Type 1 DM who began treatment within three months of diagnosis. The study observed significant improvements over a 12-month period, including the elimination of prandial insulin in all patients and basal insulin in seven. Semaglutide treatment resulted in reduced glycated hemoglobin levels from a mean of 11.7% to 5.7%, increased fasting C-peptide levels, and improved glycemic control. No serious side effects, diabetic ketoacidosis, or significant hypoglycemia were reported after dose stabilization. These findings suggest that semaglutide may help preserve β-cell function and reduce insulin dependency in early Type 1 DM, warranting further investigation through larger, randomized clinical trials [40].

*Amylin analogs* mimic the effect of amylin, a peptide hormone that is co-secreted with insulin by pancreatic beta cells. In DM patients, the secretion of amylin is impaired, which contributes to dysregulated blood glucose levels. Amylin analog peptides slow the gastric emptying, suppress glucagon release, and promote satiety, with better control of postprandial glucose spikes. Pramlintide is a synthetic analog of human amylin, used as an adjunct to insulin therapy in both Type 1 and Type 2 DM to improve postprandial glucose control. Pramlintide is particularly useful in reducing postprandial glucose highs and lows, and it has been shown to modestly reduce HbA1c levels. However, it requires multiple daily injections, which can limit patient adherence [41,42,43].

### 4.1. Glucose-Dependent Insulinotropic Polypeptide (GIP) Receptor Agonists

GIP is another incretin hormone that is secreted from the gut in response to food intake. The ineffectiveness of gastric inhibitory polypeptide (GIP)-oriented monotherapy in type 2 diabetes mellitus (T2DM) is primarily attributed to the GIP resistance, a state wherein the physiological response to GIP is significantly impaired by several mechanisms that include downregulation of GIP receptors (due to chronic hyperglycemia effect on the beta cells), impaired beta cell function (which limits the insulinotropic effects of GIP, even with functional receptors), lipotoxicity, and insulin resistance, which lack significant GIP effects on satiety and glucagon suppression [44,45,46,47].

While GIP itself has not been widely utilized as a monotherapy due to reduced efficacy in Type 2 DM, dual agonists targeting both GLP-1 and GIP receptors have been investigated. GIP receptor agonists stimulate insulin secretion in a glucose-dependent manner, similar to GLP1R agonists. However, combining GIP and GLP1R agonism may provide synergistic effects on both glucose regulation and weight loss. Tirzepatide is a dual GLP-1 and GIP receptor agonist, a new class of drug referred to as a “twincretin”. Tirzepatide has shown superior glucose-lowering effects compared to GLP1R agonists alone and has significant benefits in weight reduction, with indications in DM and for obesity management. In the SURPASS trial series, SURPASS-2 compared tirzepatide in three dose arms against semaglutide, and the most common reason for premature discontinuation of tirzepatide or semaglutide was the occurrence of adverse events, which were more common with tirzepatide at doses of 10 mg and 15 mg than with tirzepatide at a dose of 5 mg, and with semaglutide at 1 mg. The most common adverse events were gastrointestinal (nausea, diarrhea, vomiting) and were primarily mild to moderate in severity [46,48,49,50,51,52].

### 4.2. Glucagon Receptor Antagonists (GRA)

While glucagon itself is not used as a therapeutic agent in diabetes, except in some cases to overcome severe hypoglycemia, glucagon receptor antagonism has been explored as a strategy to control hyperglycemia. Glucagon promotes hepatic glucose production, and blocking its receptor can help reduce this effect, decrease hepatic glucose output, thus inhibiting an endogenous source of hyperglycemia and potentially lowering fasting blood glucose levels. Several GRA are under investigation in preclinical studies or early phase trials, but none have been widely approved for clinical use yet due to concerns about side effects and long-term safety [53,54].

A combination therapy of SGLT2 inhibitor dapagliflozin and glucagon receptor antagonist volagidemab was investigated in Type 1 DM patients, and the influence of the combination therapy on clinical outcomes such as diabetic ketoacidosis (DKA) was assessed. Euglycemic diabetic ketoacidosis (EDKA) is a form of diabetic ketoacidosis characterized by normal or mildly elevated blood glucose levels (<200 mg/dL) but with significant ketonemia and metabolic acidosis. Unlike classic ketoacidosis, EDKA lacks the hallmark hyperglycemia, making it diagnostically challenging. The condition has been increasingly associated with the use of SGLT-2 inhibitors, which promote glucose excretion in urine and elevate glucagon levels, enhancing ketogenesis. All 12 patients on dapagliflozin–volagidemab therapy used hybrid closed-loop insulin systems and were treated with the SGLT2 inhibitor alone and in combination with GRA for four weeks each. Key study outcomes included changes in glycemic control, insulin use, and ketogenesis during insulin withdrawal tests. Continuous glucose monitoring (CGM) and insulin dosing data were collected, and participants’ satisfaction and safety were assessed. The results indicated significantly improved glycemic control after combination therapy, with reduced average glycemia and increased time in the target glucose range compared to baseline and SGLT2 inhibitor monotherapy. Insulin use decreased by 27% with the combination therapy, and ketogenesis during insulinopenia was moderated, with lower peak β-hydroxybutyrate levels observed. The diminished ketogenesis that was recorded with volagidemab could indicate a metabolic shift towards decreased lipid use, thus potentially lowering EDKA incidence in the patients on combination therapy. This therapy also increased patient satisfaction without increasing hypoglycemia or other adverse events. The study concluded that adding GRA to SGLT2 inhibitor therapy could enhance the benefits of SGLT2 inhibitors while reducing the risk of DKA in Type 1 diabetes patients [55,56].

### 4.3. Multiple Receptor Agonists

A novel approach in peptide-based therapies involves the development of agents that target GLP-1, glucagon, and/or GIP receptors. These hybrid peptides, acting as multiple agonists, exploit the glucose-lowering effects of GLP-1 and GIP while stimulating the glucagon receptor to enhance energy expenditure and promote weight loss. The multiple receptor approach aims to provide both glycemic control and significant metabolic benefits. Cotadutide is one such peptide, targeting GLP-1 and glucagon receptors and showing promise for reducing glucose levels and inducing weight loss in early clinical trials. Its development was canceled in 2023 by AstraZeneca, which explored alternative agents [57,58]. Tirzepatide, the dual GLP-1 and GIP receptor agonist, has demonstrated powerful glycemic control and weight loss, with significant improvements in HbA1c levels and a notable reduction in body weight in clinical trials such as the recently completed SURMOUNT-4 trial [48,50,51].

Oxyntomodulin is a peptide hormone, discovered in 1982, that acts on both GLP-1 and glucagon receptors, influencing appetite regulation with subsequently reduced food intake, and increasing energy expenditure. Oxyntomodulin analogs act by reducing food intake and increasing energy expenditure through their combined action on GLP-1 and glucagon receptors. Several peptides are under development for the treatment of obesity and Type 2 diabetes, as they offer potential benefits in weight management and glucose control [59,60].

A phase 2, double-blind, randomized, placebo-controlled trial investigated the efficacy and safety of retatrutide, a triple hormone receptor agonist (GIP, GLP-1, and glucagon receptors), for the treatment of obesity. The study enrolled 338 adults with a body mass index (BMI) of 30 or higher or a BMI between 27 and 30 with at least one weight-related comorbidity (hypertension, dyslipidemia, or cardiovascular disease). Participants were randomized into different dose groups or placebo and received weekly subcutaneous injections for 48 weeks. The primary outcome measured was the percentage change in body weight at 24 weeks, with secondary outcomes including weight reduction at 48 weeks and improvement in cardiometabolic risk factors. All participants received lifestyle counseling, including dietary and physical activity recommendations. The results demonstrated significant weight reductions in the retatrutide groups compared to placebo. At 48 weeks, participants receiving the highest dose (12 mg) achieved an average weight loss of 24.2%, compared to a 2.1% reduction in the placebo group. Furthermore, a majority of participants achieved weight reductions of 5% or more, with higher doses showing greater efficacy. Retatrutide also improved cardiometabolic factors, including blood pressure and glycemic control, with 72% of participants with prediabetes reverting to normoglycemia. The study concluded that retatrutide is a promising candidate for obesity treatment, with significant weight loss and favorable cardiometabolic effects [61].

### 4.4. C-Peptide Therapy

C-peptide is a secondary product of insulin synthesis in the pancreatic beta cells, with anti-inflammatory and antioxidant effects in microvascular function and nerve health, which could potentially alleviate symptoms of diabetic complications like retinopathy and neuropathy and also protect against other end-organ complications. Research into C-peptide replacement therapy is ongoing, but it has not yet reached widespread clinical use [62,63,64].

### 4.5. Ghrelin Antagonists

Ghrelin is a peptide hormone that stimulates appetite, thus antagonizing its effects could help with weight management in people with DM and obesity. Ghrelin antagonists, such as the endogenous liver-expressed antimicrobial peptide 2 (LEAP-2), would block the action of ghrelin, reducing appetite and food intake, with subsequent weight loss. They also appear to have a prominent role in limiting addictive behaviors associated with substance abuse in alcohol, oxycodone, and cocaine use [65,66,67].

### 4.6. Teplizumab

Teplizumab is a humanized anti-CD3 monoclonal antibody that delays the progression of Type 1 DM and preserves pancreatic β-cell function, and was FDA approved for patients with stage 2 Type 1 DM. It targets the CD3 antigen part of the T-cell receptor complex, disrupting the activation of autoreactive CD8+ T cells that mediate β-cell destruction. Simultaneously, Teplizumab promotes the activity and preservation of regulatory T cells (Tregs), enhancing self-tolerance and mitigating autoimmunity. This dual action results in a phenotypic shift in immune cells, inducing exhaustion in pathogenic CD8+ T cells and fostering gut-tropic regulatory cells, thereby preserving residual β-cell function. Clinical trials have demonstrated that Teplizumab significantly delays T1DM onset in high-risk individuals and reduces the decline in C-peptide levels, a marker of β-cell function, in recent-onset T1DM patients. These effects lead to reduced insulin requirements and improved glycemic control, with benefits lasting up to several years post-treatment. However, its efficacy is influenced by factors such as timing of administration and patient age, with younger individuals responding more favorably. Adverse effects, including transient lymphopenia and mild cytokine release syndrome, are generally manageable. Teplizumab represents a promising advance in T1DM management, particularly as part of combination therapies aimed at prolonging β-cell survival and delaying disease progression. Future research (including the undergoing Clinical Trial NCT04598893 PROTECT extension study) is needed to optimize its long-term efficacy, minimize side effects, and refine patient selection criteria [68,69].

## 5. Insulin Pumps, Continuous Glucose Monitoring, and Automated Insulin Delivery (AID) Systems

Insulin pump technology was developed before the modern recombinant insulins. The first such devices were large, heavy, and difficult to use except in research settings. They were initially intended as a proof of concept to demonstrate the possibility of continuous insulin infusion to achieve glycemic control in diabetes patients, as an alternative to multiple daily insulin injections. The first generation of insulin pumps that had sensors or sensor-augmented pumps (SAP) were equipped with an insulin shutoff feature that activated if the user did not respond to a hypoglycemia alert. The second generation, known as the hybrid closed loop, automatically adjusts basal insulin rates while requiring manual user input for prandial boluses. The upcoming third generation, featuring fully automated multihormone closed-loop systems, is expected to fully realize the concept of an artificial pancreas [70,71,72].

The development of CGM systems in the 2000s led to the integration of insulin pumps with CGM, with significant improvement in diabetes management. CGMs measure glucose levels in real-time by analyzing the interstitial fluid and provide continuous data to the user and healthcare provider. In 2006, the MiniMed Paradigm REAL-Time System (Northridge, CA, USA) became the first commercially available insulin pump integrated with a CGM. This system enabled users to visualize their glycemic trends, helping them adjust insulin delivery more effectively. Although users still had to intervene manually, this integration marked a major leap toward automated insulin delivery [73].

Although the automated delivery systems rely on insulin, glucagon, a counter-regulatory hormone, is central to glucose and ketone metabolism, and its fluctuations complicate efforts to standardize treatment protocols. Firstly, endogenous glucagon levels can vary significantly among individuals and under different clinical scenarios, such as fasting, stress, or medication use. SGLT-2 inhibitors, often linked to EDKA, are known to elevate glucagon levels, making it hard to predict patient-specific responses. Secondly, unlike glucose or ketone levels, glucagon is not routinely monitored in clinical settings due to the lack of rapid, reliable assays, making real-time adjustments in treatment protocols impractical. Thirdly, DM patients can have differing baseline glucagon levels and responses depending on factors like insulin resistance, stress, and comorbid conditions. Furthermore, glucagon dysregulation can be associated with hyperglycemia in DM. Treatment algorithms must account for this heterogeneity, which adds complexity to creating a one-size-fits-all approach. On the other hand, exogenous glucagon can be a useful addition in dual hormone pump systems, aiming to prevent hypoglycemia, especially in the case of exercise-induced hypoglycemia during aerobic exercise when compared to single hormone pump systems [54,55,74,75].

Several recent investigations with Type 1 DM patients showcase the benefits of integrated systems in diabetes management. A case series on the use of SAP therapy in neonatal diabetes mellitus (NDM) reported on three patients with NDM who began SAP therapy with the MiniMed 640G system Northridge, California, USA() shortly after birth. All three patients showed positive outcomes with SAP therapy, particularly in stabilizing glycemic levels and preventing hypoglycemia. The use of SmartGuard Technology was found to be particularly useful for detecting and mitigating low blood glucose in the neonatal period, when frequent feeding and glucose fluctuations are common. The therapy also reduced the burden on caregivers by automatically adjusting insulin delivery and recording key data, including daily insulin doses and glucose levels, which helped in long-term management. The report also underlined the importance of monitoring for skin complications, which can arise from prolonged use of insulin pumps in neonates [76].

A second study, conducted over 3 years with 62 participants, investigated the long-term efficacy of SAP with predictive low-glucose management (PLGM) using the same system combined with telemedicine follow-up in adults with diabetes. The combination of SAP-PLGM and telemedicine led to significant reductions in HbA1c, particularly in patients with high initial HbA1c levels (Group A), as well as a substantial decrease in severe hypoglycemic events in patients prone to hypoglycemia (Group B). Telemedicine allowed for remote monitoring and better treatment adjustments. The study concluded that the integration of SAP-PLGM with telemedicine provides a highly effective strategy for managing DM in real-life settings, particularly in reducing hypoglycemic episodes and improving overall glycemic control. High compliance with sensor use was observed, supported by frequent telemedicine consultations, which facilitated personalized adjustments to insulin therapy. The findings highlight the benefits of combining advanced diabetes technologies with telemedicine to improve the long-term outcomes [77].

Time in Range (TiR) is a critical metric in diabetes therapy monitoring, defined as the percentage of time (usually over 24 h) when a patient’s glycemia remains within the target range of 70–180 mg/dL. This metric, derived from CGM, addresses limitations of the traditional HbA1c measure, which reflects average glucose levels over 2–3 months but fails to capture glycemic variability and acute glucose fluctuations. Unlike HbA1c, which can be influenced by conditions like anemia or hemoglobinopathies, TiR offers a dynamic view of daily glucose patterns, allowing for more precise adjustments to therapy and risk assessment. TiR correlates with HbA1c but provides additional insights into hypoglycemia and hyperglycemia risks. By integrating TiR with HbA1c, clinicians can better address both long-term outcomes and immediate patient safety concerns [78].

A recent prospective follow-up investigated registry data from 46,043 individuals across Germany, Austria, Switzerland, and Luxembourg. The results demonstrated a significant increase in the adoption of SAP and AID technologies, with the highest use observed in younger age groups. There was also a marked improvement in metabolic outcomes, TiR, and HbA1c levels, particularly in the pubertal and postpubertal groups. The findings revealed a significant increase in SAP and AID use, from 28.7% to 32.9% and 3.5% to 16.6%, respectively, during the study period. Younger children (<7 years) demonstrated the highest use of insulin pumps (86.1%) and AID systems (30.6%), while CGM usage was highest among children aged 7 to 11 years (90.7%). Gender differences were noted, with females using SAP more frequently than males, though no significant gender differences were observed for AID use. Notably, metabolic control, as measured by HbA1c and TiR, improved in all groups, particularly in older adolescents and young adults. The study demonstrated an increased use of diabetes technologies, particularly in younger age groups, with positive impact on glycemic control. However, the study also noted that children below 7/6 years, with low daily insulin requirements, might still rely more on SAP systems with predictive low-glucose suspend (PLGS) technology, as some AID systems were not yet optimized for this age group. The PLGS algorithms stop insulin delivery when an impending hypoglycemic episode is predicted [79].

Another recent multi-center, randomized controlled trial, with 104 participants, aimed to compare the efficacy and safety of a tubeless, on-body AID system and a tubeless, on-body SAP in adults. The participants were divided into two groups, one using the AID system (EOPatch X; Eoflow Co., Ltd., Seongnam, Republic of Korea) and the other using the SAP (EOPatch M; Eoflow Co., Ltd,, Seongnam, Republic of Korea), for a period of 12 weeks. The primary outcome was the percentage of TiR for blood glucose levels, while secondary outcomes included time above and below range (TAR, TBR), mean glucose levels, and treatment satisfaction. The results showed that the AID system significantly increased TiR from 62.1% to 71.5% compared to the SAP group, which saw a smaller increase from 64.7% to 66.9%. The AID system also outperformed the SAP in reducing TAR, TBR, and glycemic variability. HbA1c levels decreased in both groups but did not differ significantly between them. Notably, no DKA or severe hypoglycemia events occurred, indicating both systems were safe. Participants in the AID group achieved better glycemic control, especially during nighttime. The study concluded that the tubeless, on-body AID system provided superior glycemic outcomes compared to the SAP. The AID system’s benefits were consistent throughout the trial, suggesting it is an effective option for glycemic management [80].

## 6. Islet Cell Transplantation, Organoids, and Biological Pancreas Augmentation

Islet cell transplantation is a therapeutic procedure used primarily for individuals with Type 1 DM who experience severe, difficult-to-control blood glucose fluctuations or frequent episodes of hypoglycemia unawareness. The procedure involves isolating and transplanting islet cells (including insulin-producing beta cells) from a donor pancreas into the recipient, typically through the portal vein, aiming to restore the body’s ability to produce insulin and regulate blood glucose levels. The introduction of the Edmonton protocol in 2000 improved transplantation outcomes by refining donor selection and immunosuppressive regimens. Despite this progress, long-term insulin independence remains limited, as immune suppression is still required, and graft survival often diminishes over time. Recent clinical trials have demonstrated that, while transplantation reduces severe hypoglycemia and improves glycemic control, maintaining long-term insulin independence remains a challenge. Strategies for overcoming the challenges to islet cell transplantation include use of stem cell-derived islet cells and porcine islet cells. Additionally, advancements in islet encapsulation technology and co-transplantation with immunomodulatory cells, like mesenchymal stem cells (MSCs) and regulatory T cells (Tregs), are under investigation to improve islet graft survival and minimize the need for long-term immunosuppression. New transplant sites, such as the omentum and subcutaneous space, are also being explored to improve islet engraftment and reduce complications associated with the traditional portal vein infusion [81,82,83,84,85].

Cellular encapsulation is a novel approach in the treatment of diabetes, particularly for Type 1 DM. The concept involves encapsulating insulin-producing cells (such as pancreatic beta cells) in a semi-permeable membrane before transplanting them into the patient. This encapsulation serves to protect the transplanted cells from the patient’s immune system, which would otherwise attack and destroy them. Key roles of encapsulation include immune protection, as macromolecules cannot pass through the membrane, reduced need for immunosuppression due to the abovementioned effect, and effective glycemic regulation as the small molecules (glucose and regulatory molecules) can pass unhindered. While cellular encapsulation is still under development and not yet widely available, it promises improved quality of life for people with Type 1 DM by providing a more natural and long-term solution for insulin delivery [86,87].

Another approach to mitigate xenotransplanted islet cells rejection involves antibodies directed against specific immune effector mechanisms. One of the most effective individual therapies tested for preventing transplant rejection in animal models has been antibody blockade of the CD40/CD40 ligand pathway. Tegoprubart, a monoclonal antibody targeting CD40 ligand, inhibits co-stimulation, reducing cell- and antibody-mediated immunity while sparing islet cells from toxicity. Effective in animal models of islet and kidney transplantation, tegoprubart is under investigation in a calcineurin-free kidney transplant study involving up to 12 participants. Participants receive rATG induction and maintenance therapy with tegoprubart, mycophenolate, and corticosteroids, with safety as the primary endpoint. As of May 2023, five transplants have been performed, with no rejections observed. One participant discontinued due to BK viremia, the only serious adverse event reported, and another due to mild alopecia and fatigue. Tegoprubart has shown a good safety profile, no rejection episodes, and preserved graft function, supporting its evaluation in islet cell transplantation. A study to assess its efficacy in this population is planned at the University of Chicago [88,89].

A retrospective cohort study examined the relationship between primary graft function (PGF), measured 28 days post-transplantation using the Beta2-score, and five-year outcomes in 1210 Type 1 diabetes patients who underwent islet allotransplantation across 39 centers worldwide, enrolled in the Collaborative Islet Transplant Registry (CITR). The Beta2-score is a validated, continuous metric used to assess the functional performance of islet grafts following transplantation. It provides a quantifiable measure of early graft function based on a single fasting blood sample, typically collected 28 days after the last islet infusion. The score reflects the combined contribution of insulin production (derived from C-peptide levels), glucose control (evaluated by fasting glucose and HbA1c levels), and insulin independence (assessed from the need for exogenous insulin), allowing clinicians to evaluate graft success and predict long-term outcomes. Participants in the study (59.5% female, mean age 47 years) received one or more islet infusions, with a median transplanted mass of 10.8 thousand islet-equivalents per kilogram. The study evaluated the cumulative incidence of four unfavorable outcomes: unsuccessful transplantation, graft exhaustion, inadequate glucose control, and need for exogenous insulin therapy. The results demonstrated an inverse linear relationship between PGF and all outcomes, with a higher Beta2-score correlating with lower risks. For example, each 5-unit increase in PGF was associated with a 23% reduction in unsuccessful transplantation risk (adjusted subhazard ratio: 0.77). Predictive models based on PGF showed good accuracy (C-statistics: 0.65–0.76). The findings emphasized PGF’s critical role in predicting long-term outcomes, highlighting the importance of optimizing early graft function to improve success rates in islet transplantation. Limitations included the retrospective design, lack of complications analysis, and reliance on imputed data for incomplete variables. The study suggests PGF could serve as a surrogate marker for transplantation success and guide clinical decisions, such as repeated infusions [90,91].

A smaller single-center cohort study also examined long-term outcomes in 255 patients with Type 1 diabetes undergoing allogeneic islet transplantation at the University of Alberta Hospital between 1999 and 2019. The study also highlighted the importance of early graft function (Beta-2 score ≥ 15) as a predictor of sustained success and demonstrated the safety and efficacy of islet transplantation in achieving insulin independence and mitigating severe hypoglycemia, while acknowledging limitations such as retrospective design and absence of non-transplant controls. Another retrospective, multi-center study evaluated the 10-year outcomes of islet transplantation in 44 patients with Type 1 diabetes, conducted within the Swiss–French GRAGIL network between 2003 and 2010. The study also underscored the long-term safety and metabolic benefits of islet transplantation, particularly in preventing severe hypoglycemia, but highlighted the need for improved immunosuppressive protocols and strategies to enhance graft survival for better insulin independence rates. Limitations included the small cohort size, retrospective design, and missing data [92,93].

Organoids are three-dimensional (3D) cell cultures that mimic some structural and functional aspects of their tissue of origin. The organoids offer a promising platform for studying disease mechanisms and testing potential treatments. The pancreatic organoid technology is particularly suited for DM treatment. While human pluripotent stem cells have shown promise for generating insulin-secreting cells, safety concerns associated with cellular reprogramming, such as mutagenesis and carcinogenesis, remain. Organoids derived from adult pancreatic stem cells offer an alternative by reducing the risk of such complications. In DM, organoid models derived from pancreatic ductal cells have shown potential for generating insulin-producing beta cells, though this process remains inefficient, and further refinement in culture conditions is needed. Additionally, pancreatic duct-derived organoids have been studied for their ability to proliferate and differentiate into both endocrine and exocrine cells, although differentiation into functional beta cells remains limited [94,95,96,97].

The first drugs with pancreas-enhancing features were the insulin secretagogues, developed since the 1950s, which increase the beta cells insulin response to glucose levels, thereby lowering glycemia in people with Type 2 DM. The non-sulfonylurea secretagogues were introduced in the 1990s and enhance insulin secretion in a more glucose-dependent manner, which reduces the risk of hypoglycemia compared to sulfonylureas [98].

Future strategies on pancreas enhancement could involve changes that preserve beta-cell function, induce beta-cell (re)generation, and support the overall pancreas health via the following:

- therapies that stimulate the regeneration or proliferation of insulin-producing beta cells in the pancreas [38].
- beta-cell stress reduction by anti-inflammatory agents, antioxidants, and modulators of cellular stress response pathways [99].
- development of immune tolerance in Type 1 DM, with antigen-specific immune therapies boosting regulatory T cells and eliminating the reactive T-cell clones [100].
- smart secretagogues (e.g., DPP-4 inhibitors) that modulate pancreatic insulin secretion depending on the current metabolic state [101].
- preservation of islet cell vascularization by VEGF-based therapies, similar to VEGF use to stimulate vascularization in diabetic ulcers [102].
- reprogramming non-beta cells, such as alpha cells or ductal cells, into insulin-producing beta cells, in order to replenish the beta-cell population [103].
- treatment with stem-cell-derived islet tissue or activation of pancreatic endogenous stem cells [104,105].

**Figure 1:**
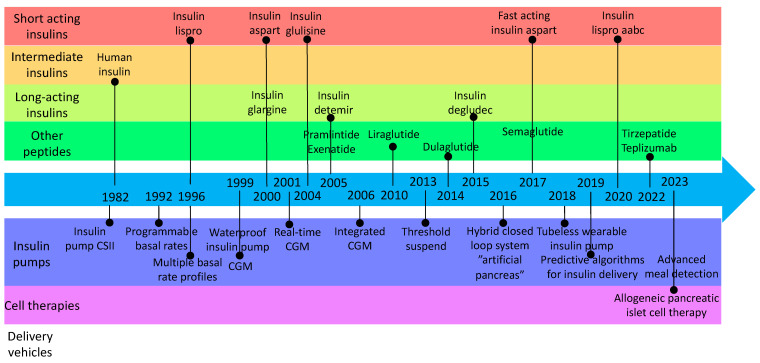
Timeline of peptide development and delivery vehicles in DM management (FDA Devices and Drugs approval dates, source: fda.gov).

On 28 June 2023, the FDA approved Lantidra (donislecel), the first allogeneic pancreatic islet cell therapy for adults with Type 1 diabetes who experience severe hypoglycemia and fail to achieve target glycated hemoglobin levels despite current management (Figure 1). Administered via infusion into the hepatic portal vein, additional doses may be given if necessary. The approval was based on two non-randomized, single-arm studies involving 30 adults, where 21 participants were insulin-independent after one year. Eleven participants maintained independence for 1–5 years, and ten for over 5 years. Adverse reactions included fatigue, anemia, and gastrointestinal symptoms, with serious effects linked to the infusion method and immunosuppression. Unlike stem cell therapy, Lantidra utilizes cadaveric islets, prompting debates about its regulatory oversight, with some organizations advocating for its management under organ transplant networks [106].

## 7. Further Biotechnology-Driven Perspectives in DM

There are clear shortcomings of recombinant or natural human insulin, which are the catalyst for future progress towards production of recombinant insulin types with delayed or faster action. Additional refinements can add metabolic sensor moieties directly on the insulin molecule-such as glucose, lactate sensors for the detection of physical activity, ketosis sensors to improve pH sensitivity-in order to modulate the hormone’s bioactivity. This would have to account for and preclude interference by other metabolites. Another possibility relies on the creation of insulin-GLP1R agonists or other types of hybrid proteins that could provide both of the parent molecules’ effects and be regulated by one or more sensors.

The advent of portable continuous glucose monitoring devices opened the way for the development of insulin pumps that could even mix various synthetic insulin types according to the patient’s current metabolic needs. Other therapeutic peptides could be added in a personalized profile for each patient’s insulin pump, depending on the clinical and biological features of the DM patient. Machine learning algorithms could be employed to predict the food habits of a patient, considering past glycemic history and current glycemic data, as well as other inputs, and tailor the pump’s response for optimal metabolic management.

Furthermore, in an engineered biological pancreas, multiple cell lines producing various types of insulin could be established, likely with islet cell glucose sensor adjustments enabling the cells to respond differently to various glycemic challenges. Thus, presumably the activation threshold for short-acting insulin would be higher than the one for long-acting insulin-producing cells.

## 8. Conclusions

Peptide-based therapies in DM have expanded significantly beyond insulin, offering various mechanisms to improve glucose regulation, promote weight loss, and reduce the risk of complications in diabetes management. GLP-1 receptor agonists, amylin analogs, and hybrid multiple receptor agonists represent some of the most important advances in this field. With ongoing research, more peptide-based therapies targeting different facets of metabolism and weight regulation are expected to emerge.

Glycemic control via the biotechnology-enabled tools would be achieved by highly selective molecular switches directly sensing interstitial molecules, through machine-learning algorithms correlating a patient’s meal habits and glycemic response history with glucose monitor real-time data and subsequent release of a personalized insulin mix or by engineered beta cells releasing potentially a combination of insulin types as they continuously sample the metabolic environment. This is most likely achieved through a combination of the above and even more.

## Figures and Tables

**Table 1:** Insulin types and their pharmacodynamic parameters [20,21,24,26,27,28,29].

Insulin	Onset of Hypoglycemic Effect	Peak Hypoglycemic Effect	Duration
Short-Acting			
Insulin aspart	15 min	1 h	2–4 h
Fast-acting insulin aspart (Fiasp)	16 min	1 h	5 h
Insulin lispro	15 min	1 h	2–4 h
Insulin lispro-aabc	15–18 min	1–2 h	2–4 h
Insulin glulisine	¼–½ h	½–1 h	2–4 h
Intermediate			
Regular human insulin	~1 h	2–4 h	5–8 h
NPH	1–2 h	4–10 h	14+ h
Long-acting, basal analogs			
Insulin detemir ^1^	3–4 h	6–8 (flattened peak)	up to 20–24 h
Insulin glargine	1.5–6 h	Peakless	24 h
Insulin degludec	0.5–1.5 h	Peakless	>42 h
Mixes			
Insulin lispro/Insulin lispro protamine 50/50	15–30 min	½–3 h	14–24 h
Insulin lispro/Insulin lispro protamine 75/25	15–30 min	½–2.5 h	14–24 h
Insulin aspart/Insulin degludec 70/30	10–20 min	1 h	>24 h
Insulin aspart/Insulin aspart protamine 70/30	15 min	Dual peak	18–24 h

^1^ To be discontinued from the US market until the end of 2024.

## Data Availability

Not applicable.
